# Bacterial lipopolysaccharide model of neuroinflammation-associated neurodegeneration in Wistar rats: A comparison between different durations of lipopolysaccharide induction

**DOI:** 10.14202/vetworld.2024.2567-2576

**Published:** 2024-11-22

**Authors:** Vandana Blossom, Sheetal Dinkar Ullal, Rajalakshmi Rai, Shrijeet Chakraborthi, Nayanatara Arun Kumar, Mangala M. Pai, Rajanigandha Vadgaonkar

**Affiliations:** 1 Department of Anatomy, Kasturba Medical College Mangalore, Manipal Academy of Higher Education, Manipal, Karnataka, India; 2 Department of Pharmacology, Kasturba Medical College Mangalore, Manipal Academy of Higher Education, Manipal, Karnataka, India; 3 Department of Cellular Pathology, Royal Preston Hospital, Fulwood, Preston, Lancashire, UK; 4 Department of Physiology, Kasturba Medical College Mangalore, Manipal Academy of Higher Education, Manipal, Karnataka, India

**Keywords:** astrocyte, lipopolysaccharide, mental health, microglia, neurodegeneration, neuroinflammation

## Abstract

**Background and Aim:**

Bacterial lipopolysaccharide (LPS)-induced neuroinflammation can be the most dependable animal model for studying neurodegeneration mechanisms driven by systemic inflammation-induced neuroinflammation. Hence, this study aimed to standardize the LPS model of neuroinflammation by comparing the effect of relatively low-dose LPS administered for different durations on the induction of neurodegeneration in Wistar rats.

**Materials and Methods:**

Six groups of six adult Wistar rats per group were used in the study. Group 1 was the control group, and the other five were administered single weekly dose of LPS (170 μg/kg) for increasing durations, ranging from 4 weeks to 8 weeks. The study endpoints included behavioral parameters, neuronal assay results, and the expression of microglia and astrocytes in the frontal cortex, dentate gyrus, and hippocampus.

**Results:**

We observed a significant reduction in the number of neurons and an increase in glial cells at 5 weeks of exposure, along with a decline in memory. Thereafter, these changes were gradual until 7 weeks of exposure. However, at 8 weeks of exposure, there was no further statistically significant worsening compared with the group exposed for 7 weeks.

**Conclusion:**

To effectively induce neuroinflammation and cause neuronal damage, a minimum of five weekly LPS administrations at a dose of 170 μg/kg is required. Moreover, our results recommend a maximum of 7 weeks of LPS exposure to create a chronic inflammatory model of neuroinflammation.

## Introduction

Neurodegenerative disorders have been on the rise worldwide in recent years, and neuroinflammation has been implicated in neurodegeneration [1, 2]. Systemic inflammation is believed to play a critical role in neuroinflammation and the progression of most neurodegenerative disorders [3]. Neuropathological and radiological studies by Morales *et al*. [4] and Schmidt *et al*. [5] have shown that neuroinflammatory responses begin well before the marked loss of neurons. Understanding the pathophysiology of neuroinflammation caused by systemic inflammation, usually by lipopolysaccharides (LPS), and developing experimental models accordingly will pave the way for more research into finding treatment options for neurodegenerative disorders. Microglial and astrocyte activation sets off the inflammatory response by releasing various proinflammatory cytokines [6]. Astrocytes also help maintain the blood–brain barrier (BBB) [7]. The BBB structure is destroyed, and microglial cells become active during the later phase of neuroinflammation, which ultimately results in astrogliosis, a crucial factor in neuronal degeneration.

Various techniques, such as ethanol, thrombin, and chronic stress, have been successfully used to induce neuroinflammation [8, 9]. However, a bacterial LPS-induced model of neuroinflammation is preferred because of the quicker induction of neuroinflammation in experimental animals [10, 11]. Many researchers have studied the effects of LPS exposure at differing doses (20–3000 μg/kg), duration (single administration to 12 weeks), and routes of administration (intraventricular, intravenous, and intraperitoneal) on neuroinflammation and neurodegeneration, particularly Alzheimer’s disease [12–14]. However, the cognitive effects of incremental duration of exposure to low-dose intraperitoneal LPS in Wistar rats have not been studied.

This study focused on a relatively low dose and widely spaced duration of intraperitoneal LPS injection. Higher LPS dosages (0.02 mg to 3 mg/kg body weight) might exert other toxic effects on the biological system of animals. This study aimed to standardize the minimum duration of weekly low-dose LPS exposure to induce optimum neuroinflammation to save time and resources and to prevent undue toxicity in Wistar rats. We compared different durations of low-dose LPS administration to induce neurodegeneration driven by neuroinflammation in an attempt to standardize an LPS model of neuroinflammation. Neuroinflammation was assessed using cognitive behavioral parameters and neuronal assays and the expression of microglia and astrocytes in the frontal cortex, dentate gyrus, and hippocampus [13].

## Materials and Methods

### Ethical approval

This study was approved by the Institutional Animal Ethics Committee of Kasturba Medical College, Mangalore (Approval number: KMC/MNG/IAEC/06/2020). The experiment was carried out in accordance with the guidelines of the Government of India for the use of laboratory animals [15].

### Study period and location

The study was conducted from January 2021 to December 2021 in the Central Animal House and Department of Anatomy at Kasturba Medical College, Mangalore.

### Animals

In-house-bred male Wistar rats, approximately 6 weeks old and weighing 180–200 g, were used in the study. The Wistar rats were fed water and food *ad libitum* and maintained in a pathogen-free environment. They were housed in polypropylene cages with paddy husk as the bedding material. Three Wistar rats were housed in each cage. The animals were acclimatized to the laboratory for a week before beginning the experiment.

### Bacterial LPS

L2880-25 mg LPSs from *Escherichia coli* and 055:B5 purified by phenol extraction (44M4004V) were procured from Sigma-Aldrich, USA.

### Experimental design

The Wistar rats (n =36) were divided into six groups (n = 6/group) as follows – Group 1: Normal control; Group 2 received 170 µg/kg body weight of LPS intraperitoneally, once a week for 4 weeks (LPS4W); Group 3 received 170 μg/kg body weight of LPS intraperitoneally, once a week for 5 weeks (LPS5W); Group 4 received 170 μg/kg body weight of LPS intraperitoneally, once a week for 6 weeks (LPS6W); Group 5 received 170 μg/kg body weight of LPS intraperitoneally, once a week for 7 weeks (LPS7W); and Group 6 received 170 μg/kg body weight of LPS intraperitoneally, once a week for 8 weeks (LPS8W). The dose of LPS was chosen from the lower range of doses, as mentioned by Zakaria *et al*. [13] and Catorce and Gevorkian [14]. At the end of the experiment, the cognitive changes of the Wistar rats were tested using behavioral models.

### Behavioral study

#### Passive avoidance test

This is a standard test in animal studies to assess learning and memory capacities [16]. The “step-through” apparatus, otherwise called the shuttle box, was used to determine the avoidance response. This structure has a dark and illuminated chamber (20.3 × 15.9 × 21.3 cm each) connected through a sliding door. Stainless steel rods of length 3.175 mm, set about 8 mm apart, constitute the floor of the chamber. The passive avoidance test was conducted on 2 consecutive days. On the testing day (2^nd^ day), the Wistar rats were allowed to explore both chambers freely for 10 min before the trial. The Wistar rats were then placed in a dark compartment. After 1 min, each animal was given an electric shock (1 mA, for 3 s) through a stainless-steel fence floor. During the training or learning trial, the Wistar rats immediately crossed over to the illuminated compartment to avoid shock. On day 2, if the Wistar rats immediately ran to the illuminated chamber from the dark chamber, they were considered normal because they remembered their exposure to shock in the dark chamber. They were considered memory-impaired Wistar rats if they showed latency to run to the illuminated chamber. When the Wistar rat did not enter the illuminated compartment within 300 s, the test was terminated, and latency was recorded as 300 s. Increased latency to enter the illuminated compartment indicates poor memory retention [17].

#### Water maze test

This sophisticated method comprehensively evaluates spatial learning and memory in Wistar rats. It was conducted in a circular pool with a diameter of 1–1.8 m and a depth of around 60 cm, which was painted with black paint. The pool was subdivided into quadrants with visual cues surrounding the pool to facilitate orientation. A hidden platform was submerged just below the surface in a quadrant. During the training phase, Wistar rats undergo multiple trials to swim to find the hidden platform, aided by visual clues placed around the room. If the user fails to locate the platform within a set time, it is gently guided to the platform. After training, probe trials were conducted in which the platform was removed, and the animals were allowed to swim freely. The time spent in the target quadrant, where the platform was previously kept, was measured. Furthermore, the time to reach the target quadrant was noted to indicate spatial memory retention and spatial learning [18].

### Histological study

#### Neuronal assay

After the behavioral tests, the animals were euthanized using ketamine overdose (120 mg/kg) and perfused with 10% formalin. The animals were secured on a dissection board to open the chest cavity and expose the heart for perfusion. Subsequently, transcardiac perfusion was performed using 100–150 mL of normal saline followed by 10% formalin at 1 mL/min. Once the animals were perfused with formalin, the brain was removed through cranial cavity dissection and transferred to a container containing 10% formalin for further fixation for approximately 48 h. Subsequently, paraffin blocks of brain tissue were prepared, and 6–7 μm thick sections of brain tissue with the hippocampal region were obtained using a rotary microtome. The sections were stained with cresyl violet. This solution was prepared by dissolving 100 mg of the stain powder in 100 ml of distilled water and then adding 5 mL of 10% acetic acid to maintain the pH between 3.5 and 3.8. The stained tissue was viewed under a light microscope (Nikon, Japan) with 20× magnification, and normal healthy neurons were counted in each area. Neurons with distorted membranes and pyknotic nuclei were considered degenerate.

### Expression of microglial cells and astrocytes

Samples that were embedded in paraffin and fixed with formalin were used for the immunohistochemistry examination. Reactive microglial cells were identified using CD68 immunohistochemistry and reactive astrocytes were identified using glial fibrillary acidic protein (GFAP) immunohistochemistry. Brain tissue was sectioned into slices of 4–6 μm thickness using a microtome. Deparaffinization and rehydration of tissue sections were achieved by sequential immersion in xylene and a graded ethanol solution. Antigen retrieval was performed by incubating the sections in citrate buffer solution (Dako, Denmark) a high temperature (80°C). Endogenous peroxidase activity was quenched using hydrogen peroxide after cooling. Non-specific binding sites were also blocked using a blocking buffer (Dako) containing normal serum. The tissue sections were then incubated overnight at 4°C with primary antibodies specific for CD68 (ES3940 Origin Diagnostic and Research, Kollam, Kerala, India) (monoclonal mouse anti-CD68 antibodies) and GFAP (ES5510 Origin Diagnostic and Research) (polyclonal rabbit anti-GFAP antibody) diluted in a blocking solution. After washing, sections were incubated with appropriate secondary antibodies conjugated to horseradish peroxidase (Dako). Immunoactivity was visualized using a diaminobenzidine chromogen solution (Dako), and counterstaining with hematoxylin was performed to visualize cell nuclei. Finally, the sections were dehydrated, cleared, and mounted in a mounting medium. Microglia undergo morphological changes during neuroinflammatory processes, ranging from oval to rod-shaped to comma-shaped [19].

### Scoring

In the subregions of the hippocampus (CA1–CA4), 250 μm long area, the frontal cortex, 300 μm^2^ area, and the dentate gyrus, 150 μm^2^ were selected for counting microglia, astrocytes, and healthy neurons. The NIS-Elements Br Software version 4.30 (www.microscope.healthcare.nikon.com) was used to count the neurons.

### Statistical analysis

Data were analyzed using a one-way analysis of variance followed by Tukey’s *post hoc* test using Jamovi 2.3.28 software (https://www.jamovi.org).

## Results

### CD68-positive microglia

**Figure-1:**
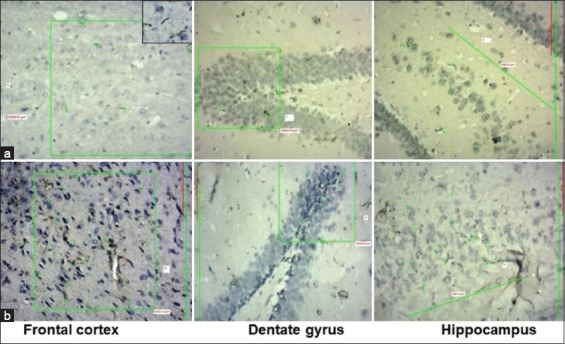
Photomicrographs of CD68-stained microglial cells in the frontal cortex, dentate gyrus, and hippocampus. (a) Control group and (b) 8-week lipopolysaccharide exposure; green star indicates rod-shaped microglia and green box indicates the area counted.

We observed a substantial increase in CD68-positive microglial cells in the groups exposed to LPS for 5, 6, 7, and 8 weeks (Figure-1). This increase rose sequentially every week till the 8^th^ week of LPS exposure. When intergroup comparisons were performed, we noted a statistically significant difference in the number of microglial cells in the dentate gyrus between all groups (Table-1). In the microglial cells of the hippocampus and frontal cortex, there was a significant increase in all groups, except between the groups exposed to LPS for 7 and 8 weeks, where the difference, though statistically not significant, was higher in the group exposed to LPS for 8 weeks.

**Table-1:** Number of microglial cells in different brain regions.

Groups	DG	CA4	CA3	CA2	CA1	FC
Control	1.66 ± 0.6	2 ± 0.5	1.83 ± 0.6	1.66 ± 0.7	1.33 ± 0.4	6.83 ± 1.7
LPS4W	3.33 ± 0.9	3.83 ± 1.06	3.83 ± 1.06	3.33 ± 0.9	3 ± 0.8	11.16 ± 1.5
LPS5W	5 ± 0.8	5 ± 0.5	4.16 ± 0.6	4.16 ± 0.8	3 ± 0.8	12.33 ± 1.4
LPS6W	8.16 ± 1.2	11.5 ± 1.1	11.83 ± 1.0	12.5 ± 0.5	8.83 ± 1.3	14.83 ± 2.2
LPS7W	10.16 ± 2.2	11.83 ± 1.3	12.83 ± 1.3	12.66 ± 1.2	10.33 ± 1.2	27 ± 1.2
LPS8W	12.83 ± 1.5	12.83 ± 2.1	13 ± 2.1	13.66 ± 1.2	11.33 ± 1.3	31.33 ± 0.9

Values are expressed as mean ± standard deviation DG: Dentate gyrus; CA: Cornu ammonis; FC: Frontal cortex; LPS4W, LPS5W, LPS6W, LPS7W and LPS8W: LPS administered for 4, 5, 6, 7, and 8 weeks, respectively

### Glial fibrillary acidic protein (GFAP)-positive astrocytes

**Figure-2:**
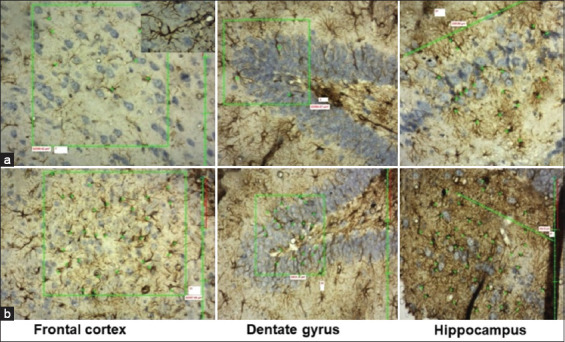
Photomicrographs of glial fibrillary acidic protein-stained astrocytes in the frontal cortex, dentate gyrus, and hippocampus. (a) Control group and (b) 8-week lipopolysaccharide exposure; green star indicates astrocytes and green box indicates the area counted.

This study showed an increase in GFAP-positive astrocytes in all LPS-exposed groups compared with the control group (Figure-2). When an intergroup comparison was performed between the LPS-exposed groups, we observed a statistically significant difference in the frontal cortex and different regions of the hippocampus (Table-2). Compared with LPS8W and LPS7W, no significant difference existed in all regions except CA3 and CA4.

**Table-2:** Number of astrocytes in different brain regions.

Groups	DG	CA4	CA3	CA2	CA1	FC
Control	3.83 ± 0.6	4.83 ± 0.8	5.83 ± 1.34	4.16 ± 1.1	3.83 ± 0.6	6.83 ± 1.7
LPS4W	7.66 ± 1.1	16.83 ± 2.4	8.66 ± 1.9	4.66 ± 1.4	5.5 ± 1.2	11.16 ± 1.5
LPS5W	8.33 ± 1.6	20 ± 1.2	11.66 ± 1.2	5.5 ± 1.5	5.8 ± 1.0	12.33 ± 1.4
LPS6W	10.5 ± 1.9	30.33 ± 1.6	12.66 ± 1.9	6.33 ± 0.8	6.5 ± 1.3	14.83 ± 2.2
LPS7W	30 ± 1.8	35.66 ± 0.7	21.16 ± 1.06	12.5 ± 0.95	16.66 ± 1.4	27 ± 1.2
LPS8W	33.16 ± 1.8	44.5 ± 1.2	26 ± 1.2	19.5 ± 0.95	23.83 ± 2.8	31.33 ± 0.9

Values are expressed as mean ± standard deviation DG: Dentate gyrus; CA: Cornu ammonis; FC: Frontal cortex; LPS4W, LPS5W, LPS6W, LPS7W and LPS8W: LPS administered for 4, 5, 6, 7, and 8 weeks, respectively.

### Neuronal assay results

**Figure-3:**
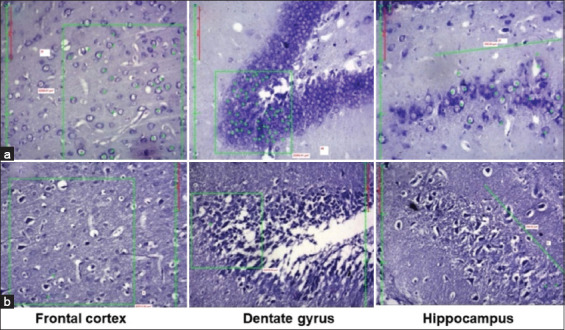
Photomicrographs of viable neurons in the frontal cortex, dentate gyrus, and hippocampus. (a) Control group and (b) 8-week lipopolysaccharide exposure; green star indicates viable, healthy neurons and the green line indicates the area selected for counting the neurons.

The study revealed a steady decline in the number of neurons in the hippocampus in all groups treated with LPS, more so in groups treated for 6, 7, and 8 weeks (Figure-3). This indicates that LPS induces neuronal damage through weekly treatment for 6 weeks. We observed significant differences between the control and all LPS groups in the neuronal assay. The intergroup comparison showed a substantial decrease in viable healthy neurons from LPS6W onset. When the LPS8W was compared to LPS7W, there was no significant decrease in the number of viable neurons in the LPS8W group (Table-3).

**Table-3:** Number of viable neurons in different brain regions.

Groups	DG	CA4	CA3	CA2	CA1	FC
Control	41.66 ± 1.8	21.5 ± 1.8	21.5 ± 1.7	25.83 ± 1.6	23.33 ± 1.4	28.16 ± 0.6
LPS4W	13.16 ± 1.4	12 ± 1.5	15.5 ± 1.5	14.5 ± 1.2	21 ± 1	19.16 ± 1.1
LPS5W	11.5 ± 0.7	8 ± 0.8	12.5 ± 1.7	13.5 ± 1.9	14 ± 1.2	16.5
LPS6W	10.66 ± 0.7	6.5 ± 1.2	6 ± 1.1	6 ± 0.5	11.83 ± 2.1	15.33 ± 1.1
LPS7W	8.5 ± 0.9	5 ± 1.0	5.5 ± 0.9	5.66 ± 1.1	6 ± 1	9.66 ± 9.6
LPS8W	5.83 ± 0.95	4 ± 0.5	4.5 ± 1.7	4.5 ± 0.9	4.5 ± 0.9	6 ± 0.1

Values are expressed as mean ± standard deviation DG: Dentate gyrus; CA: Cornu ammonis; FC: Frontal cortex; LPS4W, LPS5W, LPS6W, LPS7W and LPS8W: LPS administered for 4, 5, 6, 7, and 8 weeks, respectively.

### Passive avoidance test

**Figure-4:**
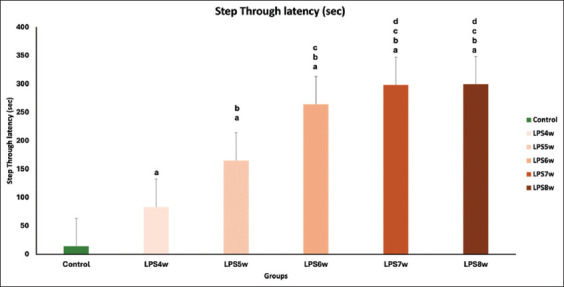
Comparison of step-through latency among the groups where a represents the control versus LPS administered for 4 weeks (LPS4W), 5 weeks (LPS5W), 6 weeks (LPS6W), 7 weeks (LPS7W), and 8 weeks (LPS8W); p < 0.001; b represents LPS4W compared with LPS5W, LPS6W, LPS7W, and LPS8W, p < 0.001; c represents LPS5W compared with LPS6W, LPS7W, and LPS8W; p < 0.001; d represents LPS6W compared with LPS7W and LPS8W; p < 0.001.

A dose-dependent increase in step-through latency indicates that memory in LPS-treated Wistar rats worsens every week. The step-through latency reached a maximum of 300 s after 5 weeks of LPS administration. This shows that LPS induces neuronal damage on weekly treatment for at least 5 weeks. When the control group (14) was compared with the LPS4W (83.33), LPS5W (165.16), LPS6W (264.33), LPS7W (297.83), and LPS8W (299.33), we observed a marked increase in the latency to enter the dark chamber, which was statistically very significant (p < 0.001). Similarly, when the intergroup comparison was performed, there was a significant increase (p < 0.001) in the latency between the groups. However, between LPS7W and LPS8W, there was no statistically significant change in the latency (p = 0.204) of entering the dark chamber (Figure-4).

### Water maze test

#### Time taken to reach the target quadrant

**Figure 5:**
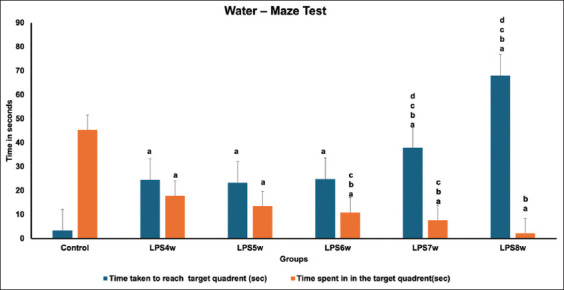
Comparison of water maze test results across the groups where a represents the control vs. LPS administered for 4 weeks (LPS4W), 5 weeks (LPS5W), 6 weeks (LPS6W), 7 weeks (LPS7W), and 8 weeks (LPS8W); p < 0.001; b represents LPS4W compared with LPS5W, LPS6W, LPS7W, and LPS8W, p < 0.001; c represents LPS5W compared with LPS6W, LPS7W, and LPS8W; p < 0.001; d represents LPS6W compared with LPS7W and LPS 8W; p< 0.001.

When the control group (13.33) was compared with LPS4W (24.5), LPS5W (23.33), LPS6W (24.83), LPS7W (37.83), and LPS8W (68), we observed a marked increase (p<0.001) in the latency to reach the target quadrant (Figure-5). When the intergroup comparison was performed, a slight increase was observed in LPS4W, followed by LPS5W. However, a significant increase was observed in LPS7W and LPS8W. However, when the LPS7W and LPS8W groups were compared, we observed a substantial increase (p < 0.0001) in reaching the target quadrant.

#### Time spent in the target quadrant

When the control group (45.33) was compared with LPS4W (17.83), LPS5W (13.5), LPS6W (10.8), LPS7W (7.66), and LPS8W (2.21), we observed a marked decrease in the time spent in the target quadrant (p < 0.001). The intergroup comparison showed no significant difference between the LPS4W and LPS5W groups. However, the LPS6W, LPS7W, and LPS8W groups significantly decreased the time spent in the target quadrant compared with LPS5W (p < 0.001). When the LPS7W and LPS8W groups were compared, no significant difference was observed in the time spent in the target quadrant.

## Discussion

**Figure-6:**
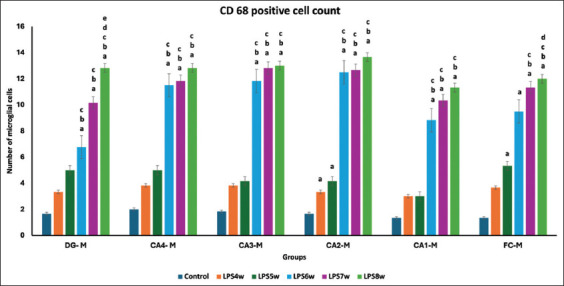
Comparison of CD68-positive astrocytes in the hippocampus and frontal cortex among groups where a represents the control versus LPS administered for 4 weeks (LPS4W), 5 weeks (LPS5W), 6 weeks (LPS6W), 7 weeks (LPS7W), and 8 weeks (LPS8W); p < 0.001; b represents LPS4W compared with LPS5W, LPS6W, LPS7W, and LPS8W, p < 0.001; c represents LPS5W compared with LPS6W, LPS7W, and LPS8W; p < 0.001, and d represents LPS6W compared with LPS7W and LPS 8W; p < 0.001.

Although various researchers have used bacterial LPS to induce neuroinflammation in experimental Wistar rats [13, 14, 20], the possible lower dosage of LPS needed to induce optimal neuroinflammation that can produce neurodegeneration and cognitive impairment, along with activation of microglia and astrocytes has not been sufficiently reported in the literature. LPS is a glycolipid that constitutes the outer membrane component of Gram-negative bacteria and provides integrity to the bacterial cell [21]. It comprises three components: Lipid A, O-antigen, and polysaccharide. Among these three, lipid A is the most bioactive and powerful component of the *E. coli*-derived LPS and is responsible for more severe inflammation than other types [22]. It induces inflammation through toll-like receptor-4 (TLR-4) and, through a complex pathway, promotes the activation of nuclear factor-kappa B, a redox-sensitive transcription factor for generating proinflammatory cytokines. Gut microbial dysbiosis or imbalance releases excessive LPS, which in turn leaks from the intestinal mucosa into the systemic circulation [10]. Interactions with TLR-4s are responsible for a cascade of pro-inflammatory reactions and the release of cytokines both in the periphery and central nervous system (CNS) through the gut-brain axis [3]. Massive host inflammatory reactions are triggered when significant amounts of LPS are released into the bloodstream, thereby endangering multiple organs. The inflammatory response is triggered by the activation of microglial and astrocytes in the CNS, which release various proinflammatory cytokines [6]. TLR-4 is widely expressed in microglia, and an increase in microglial activation may lead to neuronal damage due to a surge in proinflammatory cytokines [23]. The increase in the number of microglial cells in the brains of LPS-exposed Wistar rats (Table-1; Figures-1 and 6) indicates the augmentation of the inflammatory process by LPS. Although an increase in microglia was observed in all LPS-exposed Wistar rats, this was significantly higher in Wistar rats exposed to 7 weeks of LPS injection.

**Figure-7:**
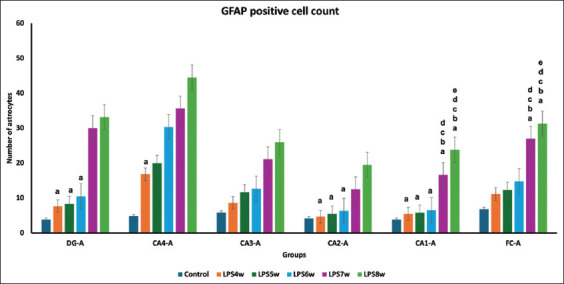
Comparison of glial fibrillary acidic protein-positive astrocytes in the dentate gyrus, hippocampus, and frontal cortex among groups where a represents the control versus LPS administered for 4 weeks (LPS4W), 5 weeks (LPS5W), 6 weeks (LPS6W), 7 weeks (LPS7W), and 8 weeks (LPS8W); p < 0.001; b represents LPS 4W compared with LPS5W, LPS6W, LPS7W, and LPS8W; p < 0.001; c represents LPS5W compared with LPS6W, LPS7W, and LPS8W; p < 0.001; d represents LPS6W compared with LPS7W and LPS8W; p < 0.001; e represents LPS7W compared with LPS8W; p < 0.001.

Astrocytes, abundant glial cells in the CNS, contribute mainly to the maintenance of BBB and other functions [24, 25]. However, these astrocytes become highly reactive in response to certain noxious stimuli. These reactive astrocytes may exert harmful effects on the CNS [26], and in neurodegenerative diseases, the number of astrocytes is alarmingly increased in the brain [27, 28]. In the present study, the number of astrocytes increased in all LPS-induced Wistar rats, and the increase was significantly higher in Wistar rats at 7 weeks of LPS injection (Table-2; Figures-2 and 7). The microglia activated by LPS-induced inflammation facilitate the conversion of dormant astrocytes to reactive astrocytes, which in turn accelerates neurodegeneration. The gradual decline in the numbers of healthy viable neurons in the frontal cortex, dentate gyrus, and all subregions of the hippocampus in LPS-induced Wistar rats indicates increased neurodegeneration. The decline in viable and healthy neurons was highly significant in the 7-week model. This increased neurodegeneration agrees with an increase in the numbers of microglia and astrocyte expressions.

In addition, the animals’ memory performance in passive avoidance (Figure-4) and water maze (Figure-5) tests declined, and all groups showed different degrees of memory deficit. Among the different durations of LPS exposure, the 7-week group showed a substantial memory deficit compared with the other groups.

**Figure-8:**
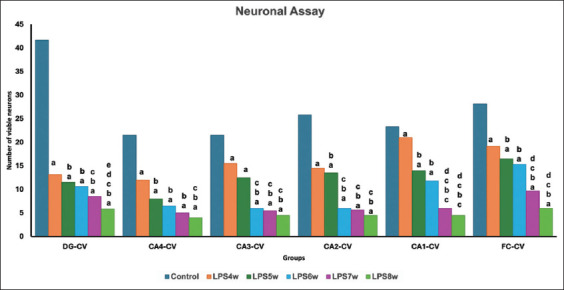
Comparison of viable neurons in the dentate gyrus, hippocampus, and frontal cortex among groups where a represents the control versus LPS administered for 4 weeks (LPS4W), 5 weeks (LPS5W), 6 weeks (LPS6W), 7 weeks (LPS7W), and 8 weeks (LPS8W); p < 0.001; b represents LPS4W compared with LPS5W, LPS6W, LPS7W, and LPS8W, p < 0.001; c represents LPS5W compared with LPS6W, LPS7W, and LPS8W; p < 0.001; d represents LPS6W compared with LPS7W and LPS8W; p < 0.001; e represents LPS7W compared with LPS8W; p < 0.05.

A study by Zhao *et al*. [3] has shown that LPS-induced neuroinflammation leads to cognitive impairment in mice with 500–750 μg of LPS. This cognitive deficit in the form of memory loss can be attributed to neurodegeneration in the hippocampus, which is a brain region mainly associated with memory. Qin *et al*. [29] and Daulatzai *et al*. [30] demonstrated that 5 mg/kg of LPS was needed to induce neuroinflammation. Other studies by Zakaria *et al*. [13], Skrzypczak-Wiercioch and Salat [20], and Wiesner *et al*. [31] have reported neuroinflammation following LPS injection, with dosages ranging from 0.02 mg to 3 mg/kg in body weight. There is a suggestion that the effects of repeated exposure to LPS on inflammatory markers in the brain are subdued, probably because of tolerance [32]. However, neurodegeneration was gradually established in this study due to neuroinflammation by the 7^th^ week. Although the study by Rahman *et al*. [16] has investigated durations ranging from 3 weeks to 12 weeks of LPS exposure, there is no report on the specific duration of LPS exposure needed to induce optimum neurodegeneration that can be substantiated by cognitive changes and neuronal counts in the key brain regions of learning and memory, namely the frontal cortex and hippocampus. The present study showed that once-weekly a relatively low dose of 170 μg/kg LPS intraperitoneally for a minimum of 5 weeks can induce slight cognitive decline, as shown in passive avoidance and water maze tests, which was confirmed by a significantly reduced neuronal count in the frontal cortex and hippocampus (Table-3; Figures-3 and 8). These effects gradually increased, and the animals exposed to 7 weeks of LPS showed significantly higher values for almost all parameters. There is no need to continue administration of LPS for more than 7 weeks as no further statistically significant worsening of cognition or neuroinflammation was observed in animals exposed to LPS for 8 weeks.

The strength of this study is that it provides a standardized method to induce chronic neuroinflammation, which can produce substantial neurodegeneration in Wistar rats, with a low dose of peripherally administered LPS. The study followed a duration escalation method that also provides a ceiling duration (7 weeks) for the induction of chronic neuroinflammation. However, further studies with a similar methodology are needed to standardize doses and duration of LPS administration in other species, especially mice, as alternate models of neuroinflammation.

## Conclusion

The results of our study suggest that a 7-week intraperitoneal injection of 170 μg/kg LPS (single dose/week) can be a reliable choice for animal models of neurodegeneration in future studies. The difference between 7^th^ and 8^th^ weeks of LPS induction was not significant, and the changes observed in the 7^th^ week were sustained in the 8^th^ week. The number of healthy neurons was significantly decreased in all regions of the hippocampus, frontal cortex, and DG. Although cognitive decline and more microglia and astrocytes were observed in the 5^th^ week, the difference was highly significant in 7^th^ week of LPS injection. In addition, the number of healthy neurons in the frontal cortex, dentate gyrus, and hippocampal subregions was significantly lower on a 7-week exposure with no further worsening on an 8-week exposure compared with other groups of shorter LPS treatment, which is suggestive of neurodegeneration.

## Authors’ Contributions

VB: Study design, data collection, analysis, and interpretation, and drafted the manuscript. SDU: Conception, study design, supervision, interpretation of results, and manuscript preparation. RR: Study design, supervision, data analysis and interpretation, and manuscript preparation. SC: Study design, data collection, and supervision. NAK: Study design, data collection and analysis, and supervision. MMP and RV: Interpretation of results, supervision, and edited the manuscript. All authors have read and approved the final manuscript.
